# Peppers in Diet: Genome-Wide Transcriptome and Metabolome Changes in *Drosophila melanogaster*

**DOI:** 10.3390/ijms23179924

**Published:** 2022-09-01

**Authors:** Carlos Lopez-Ortiz, Mary Edwards, Purushothaman Natarajan, Armando Pacheco-Valenciana, Padma Nimmakayala, Donald A. Adjeroh, Cristian Sirbu, Umesh K. Reddy

**Affiliations:** 1 Department of Biology, Gus R. Douglass Institute, West Virginia State University, Institute, WV 25112, USA; 2 Lane Department of Computer Science and Electrical Engineering, West Virginia University, Morgantown, WV 26506, USA; 3 Charleston Area Medical Center, Institute for Academic Medicine, Charleston, WV 25304, USA; 4 Department of Behavioral Medicine and Psychiatry, West Virginia University School of Medicine, Charleston Division, Charleston, WV 25304, USA

**Keywords:** habanero pepper, diet, metabolome, transcriptome, Drosophila

## Abstract

The habanero pepper (*Capsicum chinense*) is an increasingly important spice and vegetable crop worldwide because of its high capsaicin content and pungent flavor. Diets supplemented with the phytochemicals found in habanero peppers might cause shifts in an organism’s metabolism and gene expression. Thus, understanding how these interactions occur can reveal the potential health effects associated with such changes. We performed transcriptomic and metabolomic analyses of *Drosophila melanogaster* adult flies reared on a habanero pepper diet. We found 539 genes/59 metabolites that were differentially expressed/accumulated in flies fed a pepper versus control diet. Transcriptome results indicated that olfactory sensitivity and behavioral responses to the pepper diet were mediated by olfactory and nutrient-related genes including gustatory receptors (*Gr63a*, *Gr66a*, and *Gr89a*), odorant receptors (*Or23a*, *Or59a*, *Or82a*, and *Orco*), and odorant-binding proteins (*Obp28a*, *Obp83a*, *Obp83b*, *Obp93a*, and *Obp99a*). Metabolome analysis revealed that campesterol, sitosterol, and sucrose were highly upregulated and azelaic acid, ethyl phosphoric acid, and citric acid were the major metabolites downregulated in response to the habanero pepper diet. Further investigation by integration analysis between transcriptome and metabolome data at gene pathway levels revealed six unique enriched pathways, including phenylalanine metabolism; insect hormone biosynthesis; pyrimidine metabolism; glyoxylate, and dicarboxylate metabolism; glycine, serine, threonine metabolism; and glycerolipid metabolism. In view of the transcriptome and metabolome findings, our comprehensive analysis of the response to a pepper diet in Drosophila have implications for exploring the molecular mechanism of pepper consumption.

## 1. Introduction

Recently, fruits and vegetables such as tomatoes, teas, berries, and citrus have been recognized as natural sources of various bioactive compounds such as lycopene, epigallocatechin gallate, resveratrol, and flavonoids, respectively [1,2,3,4]. These natural compounds possessing multiple health benefits and relatively low toxicity have been used as a health supplement to treat diverse metabolic-related disorders [5]. Pepper (*Capsicum* spp.) represents an important crop worldwide that is used as a flavoring spice and is prominent in diets of various communities and cultures because of the beneficial properties of the phytochemicals including capsaicinoids, carotenoids, phenolic compounds, vitamins, and minerals [6,7]. Among the five domesticated *Capsicum* species, *Capsicum chinense*, which is native to Central and Southern America, has been recognized as the hottest chili in the world because of its high capsaicin content [8].

Capsaicin is the major active compound in pepper and has been found to have beneficial roles in humans, including managing pain inflammation during rheumatoid arthritis and treating non-allergic rhinitis [9,10]. Furthermore, capsaicin has been found to be an anti-cancer agent by generating reactive oxygen species and increasing apoptosis [11,12]. Similarly, because of its antioxidant properties, capsaicin has been related to the prevention or treatment of neurodegenerative diseases such as Alzheimer’s disease [13,14]. Carotenoids are another bioactive compound found in peppers responsible for fruit color; both carotenoids and capsaicin have been found to have anti-obesity effects during dietary consumption by promoting fatty acid oxidation and regulating appetite and satiety, respectively [15,16].

Diet plays a central role in organism development and fitness, and given the complexity of the whole-body response to dietary changes [17], invertebrate model organisms can serve as useful tools to examine the interplay between genes, signaling pathways, and metabolism [18]. Drosophila, an invertebrate model with its extensively studied genome, has more than 70% gene homology to humans [19]. The similarity of metabolic pathways between Drosophila and mammals has encouraged the use of Drosophila in the context of screening and evaluating the impact of different diets [20,21].

In this work, we examined the metabolomic and transcriptional response to a habanero-pepper diet in *D. melanogaster* to better understand the molecular and physiological changes associated with dietary pepper consumption.

## 2. Results

### 2.1. Phytochemical Content

To determine the phytochemical composition of the habanero pepper supplemented in the diet for Drosophila flies, we estimated the content of flavonoids, carotenoids, and capsaicinoids (Table S1). Among the phenolic compounds, apigenin had the highest content, at 110.7 µg∙g^−1^, followed by quercetin, luteolin, and kaempferol, at 40.7, 16.8, and 12.5 µg∙g^−1^, respectively. Similarly, for carotenoids compounds, the content of α-carotene and β-carotene was the highest, at 113.1 and 128.6 µg∙g^−1^, respectively. The total capsaicin content was 7828.4 µg∙g^−1^ and dihydrocapsaicin was 3836.1 µg∙g^−1^.

### 2.2. Food Intake and Phenotypic Profile of Flies on the Habanero-Pepper Diet

**Figure 1:**
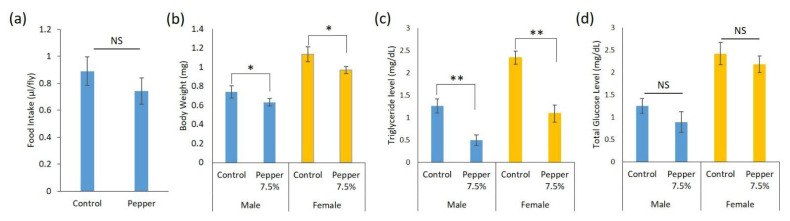
Food intake and physiological changes of *Drosophila melanogaster* in response to control and pepper diet. (**a**) Food consumption (µL/fly) (**b**) body weight determination (**c**) triglycerides content and (**d**) glucose level under control and habanero-pepper diets. Data are mean ± SEM for *n* = 10 independent replicates. *, *p* ≤ 0.05, **, *p* ≤ 0.01, NS, not significant, compared to control treatment.

The CAFE (capillary feeder) assay was used to measure food consumption in flies reared on control and pepper diets (Figure 1a). The results showed that total food consumption in the control diet was 0.89 µL/fly, while in the habanero-pepper diet was 0.74 µL/fly. Although a slight decrease in the total food consumption was observed in the pepper diet, this reduction was not statistically significant when compared to the control diet (*p* > 0.05).

Moreover, physiological effects such as body weight, triglyceride (TG), and glucose levels were determined on 5-day old adult flies reared on control and habanero-pepper diets. Flies fed a pepper-supplemented diet showed significant body-weight reduction for both sexes (*p* = 0.05) as compared with those fed with the control diet (Figure 1b). Likewise, we observed a significant (*p* = 0.01) reduction in TG level in female and male flies reared on a habanero-pepper diet (Figure 1c). Additionally, we measured the whole-body free glucose levels because obesity also affects glucose metabolism [22]. Although the glucose levels of flies reared on the pepper diet and control diet did not significantly differ, we noticed a slight reduction for both sexes (Figure 1d).

### 2.3. Metabolome Analysis of Flies on the Habanero-Pepper Diet

**Figure 2:**
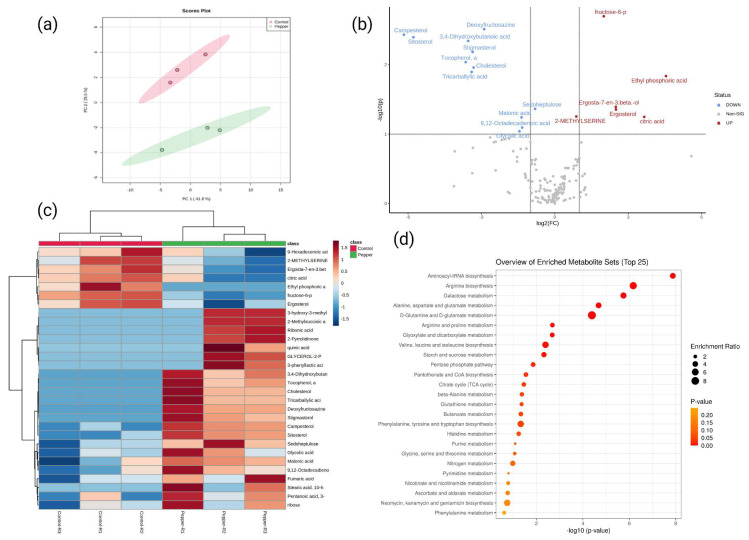
Untargeted metabolomics of *D. melanogaster* reared on a habanero-pepper diet. (**a**) 2-D principal component analysis score plot demonstrating statistical clustering of metabolites in response to habanero-pepper (green) and control (red) diet. (**b**) Volcano plot of metabolites with a fold-change threshold (x) 1.0 and *t*-test threshold (y) 0.1. The red circles represent features above the threshold. The further the position from the (0,0), the more significant the metabolite. (**c**) Heat map of the top 30 metabolites showing contrasting abundance between the two treatment diets. The red color represents upregulated metabolites and blue circles downregulated metabolites. (**d**) KEGG pathway enrichment comparison of significant metabolites found in *Drosophila* in response to a habanero-pepper diet. The y-axis indicates the term categories, the x-axis the rich factor, the size of the dot the number of metabolites found in one specific pathway, and the color of the dots the *p*-value of enrichment analysis.

To investigate the main metabolic changes reflecting the variation in diet, we used a widely targeted metabolome method to quantify primary and secondary metabolites in flies supplemented with the habanero-pepper diet. Samples from adult flies were subjected to GC-MS analysis, which resulted in the identification of 187 metabolites showing different concentrations between diets (Table S2). Principal component analysis revealed that control and habanero-pepper diets were clearly separated in the PC1 × PC2 score plot; the first two principal components accounted for 67.2% of the overall variability, with good agreement between the three replicates of each diet (Figure 2a). Metabolites identified were mainly in different classes such as amino acids, monosaccharides, carboxylic acids, fatty acids, amines, and neurotransmitters (Table S3). Moreover, the levels of 59 metabolites were markedly changed with the pepper versus control diet: 19 metabolites were downregulated and 40 were upregulated (Table S4). Among the differentially accumulated metabolites, campesterol, sitosterol, sucrose, oxamic acid, and α-tocopherol were highly upregulated with the habanero-pepper diet, and azelaic acid, ethyl phosphoric acid, citric acid, ergosterol, and fructose-6-phosphate were the major metabolites downregulated (Figure 2b,c). Furthermore, we analyzed the top 25 metabolic pathways with the largest number of differentially accumulated metabolites (Figure 2d and Figure S1) and found that the metabolites were involved in pathways such as aminoacyl-tRNA biosynthesis; arginine biosynthesis; galactose metabolism; alanine, aspartate, and glutamate metabolism; D-glutamine and D-glutamate metabolism; arginine and proline metabolism; glyoxylate and carboxylate metabolism; valine, leucine and isoleucine biosynthesis; starch and sucrose metabolism; and pentose phosphate.

**Figure 3:**
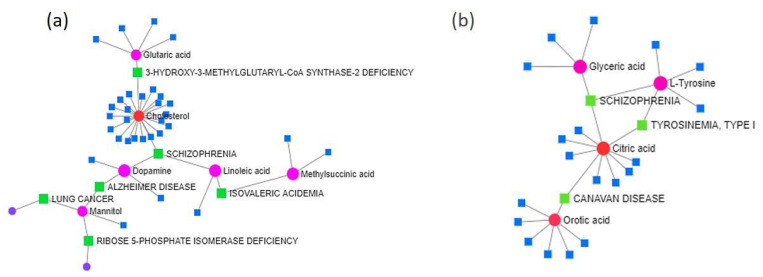
Metabolite–disease interaction network analysis of the top upregulated (**a**) and downregulated (**b**) metabolites in *Drosophila* under a habanero-pepper diet. Enriched terms are represented as nodes, and the node size represents the significance for each term. Circles represent one metabolite and squares an associated disease.

Furthermore, using the Network Explorer module from MetaboAnalyst 5.0, we created two Metabolite-Disease Interaction Networks to identify connections that cross pathway boundaries between significant up- and downregulated metabolites. For example, Figure 3a shows the plot of the created interaction network for the upregulated metabolites. In this network, compounds such as cholesterol and glutaric acid affected 3- hydroxy3-methylglutaryl-CoA synthase-2 deficiency. However, cholesterol, dopamine, and linoleic acid seemed to have a positive interaction with schizophrenia. Moreover, both, linoleic acid and methylsuccinic acid were involved in isovaleric acidemia, and the compound mannitol by itself was involved in diseases such as Alzheimer’s disease, lung cancer, and ribose-5-phosphate isomerase deficiency. Similarly, we obtained a plot using the downregulated metabolites (Figure 3b). This network showed that citric acid and orototic acid were involved in Canavan disease, whereas citric acid, glyceric acid, and L-tyrosine were involved in schizophrenia; L-tyrosine and citric acid were involved in tyrosinemia type 1.

### 2.4. Transcriptome Analysis of Flies on the Habanero-Pepper Diet

**Figure 4:**
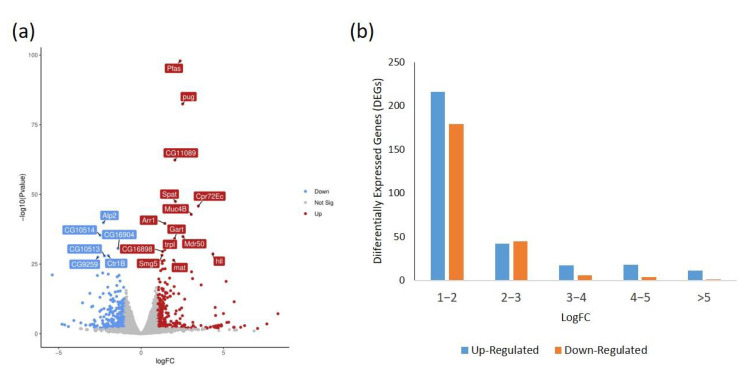
Gene expression changes in Drosophila on habanero-pepper versus control diet. (**a**) Volcano plot showing the Log2 fold change (FC) of differentially expressed genes. The Log2FC is plotted on the x-axis and the *p*-value on the y-axis. (**b**) Distribution of fold-change of gene expression in *Drosophila* on a habanero-pepper versus control diet. Down: downregulated; Up: upregulated.

To obtain a comprehensive overview of the transcriptome changes in response to the habanero-pepper diet and its effect on Drosophila, we constructed cDNA libraries for male and female adult flies reared on the pepper and control diets. These samples were paired-end sequenced by using an Illumina NextSeq500 platform. We obtained 183,528,950 raw reads, which were further filtered to produce 157,504,046 clean reads (85.81%). About 95.54% to 97.61% of the clean libraries were mapped to the Drosophila reference genome (Table S5). We identified 539 DEGs with ≥2-fold change in expression with the habanero-pepper versus control diet: 304 and 235 were up- and downregulated, respectively (Figure 4 and Table S6).

### 2.5. Functional Annotation and Classification of DEGs

The DEGs were annotated by using the BLASTx algorithm and non-redundant protein database at NCBI. Gene annotation and GO enrichment analysis was performed with BLAST2GO. The DEGs were classified into 49 categories (Figure S2). In the biological process category, “cellular amino acid metabolic process”, “alpha-amino acid metabolic process”, “cellular amino acid biosynthetic process” and “pigment biosynthetic process” terms were the most abundant. In the cellular category, “extracellular region”, “extracellular region part”, “extracellular space” and “external encapsulating structure” terms were the top four categories. Finally, in the molecular function category, the main categories were “peptidase activity, acting on L-amino acid peptides”, “peptidase activity”, “cofactor binding” and “endopeptidase activity”.

### 2.6. Pathway Analysis of DEGs

**Figure 5:**
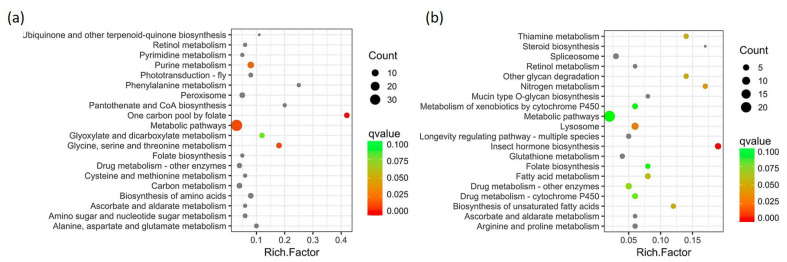
Scatter plot of top 20 enriched KEGG pathways among upregulated (**a**) and downregulated (**b**) DEGs in habanero-pepper versus control diet. The rich factor is the ratio of the number of DEGs to total gene number in a pathway. The Q-value is a corrected *p*-value. The color and size of the dots represent the range of q-values and the number of DEGs mapped to the indicated pathways, respectively.

Pathway analysis of DEGs involved using the Kyoto Encyclopedia of Genes and Genomes (KEGG) pathway database with KOBAS. The DEGs upregulated (304) and downregulated (235) with the pepper diet were assigned to 54 and 41 pathways, respectively (Tables S7 and S8). The top 20 enriched KEGG pathways among upregulated and downregulated DEGs with the pepper versus control diet are shown in Figure 5. The pathways enriched in upregulated genes with the pepper diet were “One carbon pool by folate”, “Metabolic pathways”, “Glycine, serine and threonine metabolism”, “Purine metabolism”, “Glyoxylate and dicarboxylate metabolism”, “Biosynthesis of amino acids”, and “Amino sugar and nucleotide sugar metabolism”, whereas enriched pathways for downregulated genes were “Insect hormone biosynthesis”, “Nitrogen metabolism”, “Thiamine metabolism” “Other glycan degradation”, “Biosynthesis of unsaturated fatty acids”, “Fatty acid metabolism”, “Drug metabolism-cytochrome P450”, “Metabolism of xenobiotics by cytochrome P450” “Folate biosynthesis”, “Arginine and proline metabolism” and “Longevity regulating pathway—multiple species”.

### 2.7. Functional Network Analysis of DEGs

**Figure 6:**
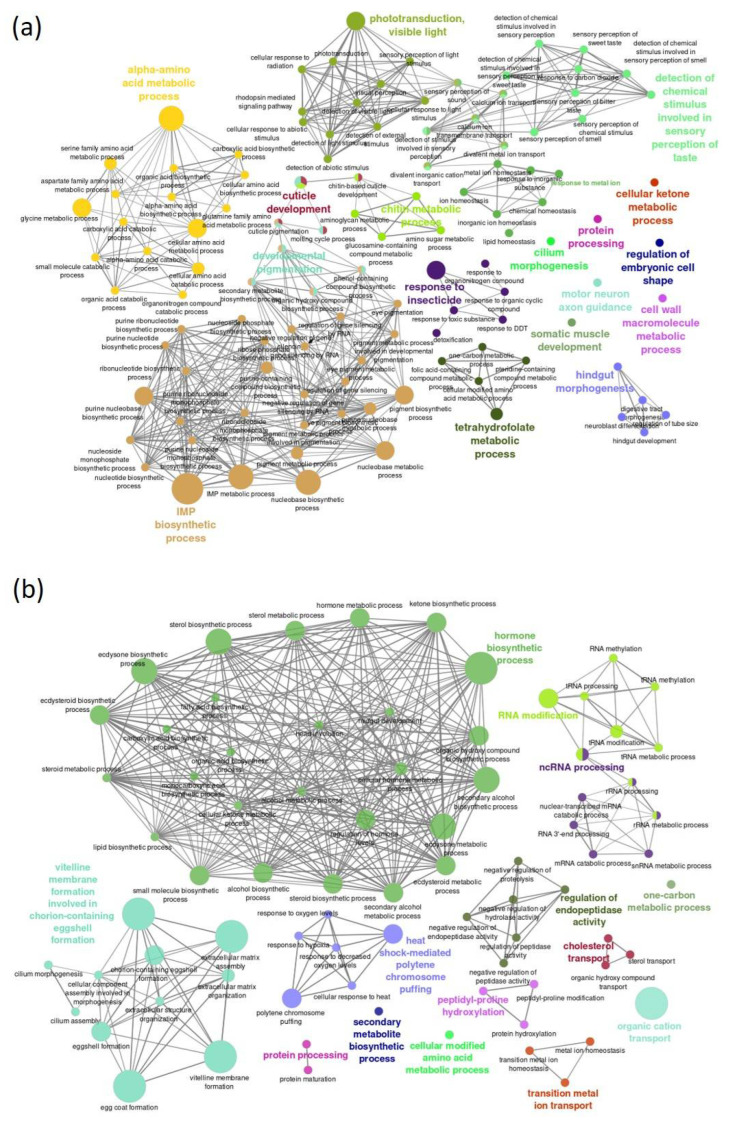
Functional network analysis of upregulated (**a**) and downregulated (**b**) genes of Drosophila with the habanero-pepper diet. The genes are functionally grouped in terms with nodes linked based on their kappa score (≥0.3); only the label of the most significant term per group is shown.

Biological gene network analysis of up- and downregulated genes involved using Cytoscape. For the upregulated genes, 17 functional network clusters were obtained, including inosine monophosphate biosynthetic process, alpha-amino acid metabolic process, phototransduction visible light, detection of chemical stimulus involved in sensory perception of taste, tetrahydrofolate metabolic process, chitin metabolic process, cuticle development, developmental pigmentation and response to insecticide (Figure 6a). Moreover, for downregulated genes, we found 14 functional networks including hormone biosynthetic process, vitelline membrane formation involved in chorion-containing eggshell formation, heat shock-mediated polytene chromosome puffing, regulation of endopeptidase activity, cholesterol transport, RNA modification and ncRNA processing (Figure 6b).

### 2.8. DEGs in Response to Capsaicin

Capsaicin is the primary bioactive substance in red chili peppers that produces the pungent flavor. We identified 12 olfactory-related genes divided into three main categories such as gustatory receptors (*Gr63a*, *Gr66a*, and *Gr89a*), odorant receptors (*Or23a, Or59a, Or82a,* and *Orco*), and odorant-binding proteins (*Obp28a, Obp83a, Obp83b, Obp93a*, and *Obp99a*). Ten of these genes were upregulated, and *Obp99a* and *Or59a* were downregulated with the habanero-pepper diet. Moreover, we obtained the human orthologues that were previously reported in response to capsaicin by using Flybase (https://flybase.org/ accessed on 1 February 2022) (Table 1). As a result, we identified that the gene inactive (*iav*), (encoding a protein involved in the response to different stimuli such as startle, heat, or sound), is an orthologue of transient receptor potential cation channel subfamily V (TRPV) proteins, including *TRPV1*, in humans. The gene *iav* showed high expression with the pepper versus the control diet. Additionally, we identified three downregulated heat shock proteins (Hsps) related to heat stress (*Hsp70Ab*, *Hsp70Aa*, and *Hsp70Bbb*). Moreover, *Muc68Ca* and *Muc30E*, which were upregulated, are orthologues of *Muc2* in humans. Downregulated *CG2839* is an orthologue of *Reg3g*. *Muc2* and *Reg3g* have been found to protect the gastrointestinal tract and modulate intestinal hormones, respectively. We identified two downregulated genes involved in fatty acid metabolism: *CG31661*, an orthologue of *PGC1-α*, was predicted to enable aspartic-type endopeptidase activity, and *FASN3*, an orthologue of FASN, encodes a fatty acid synthase that is directly related to triglyceride metabolism. Likewise, we found three upregulated genes linked to lipid metabolism: *Akh*, which encodes an adipokinetic hormone, and *Lpin*, which encodes a protein that plays a central role in fat body function and energy metabolism. Furthermore, three upregulated genes were related to environmental and metabolic stress (*Thor*, *trpl*, and *Ets21C*), while Limostatin (*Lst*) and sugarbabe (*sug*), associated with response to starvation, were up- and downregulated, respectively.

### 2.9. Integrated Metabolomic and Transcriptomic Analyses

**Figure 7:**
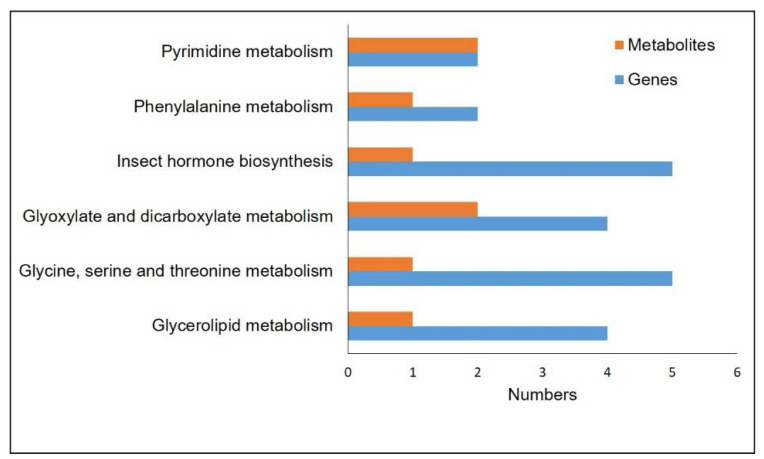
Integrated analyses of the transcriptome and metabolome on the KEGG pathways with the habanero-pepper diet. The specific KEGG pathway is labeled on the left, and the number of relative DEGs and differentially accumulated metabolites are on the right.

To further explore the association between gene expression and metabolite accumulation in Drosophila with the habanero-pepper diet, we selected differentially accumulated metabolites and DEGs in KEGG pathways for integrated analysis. We identified six changed KEGG pathways related to “Glycerolipid metabolism”, “Glycine, serine, and threonine metabolism”, “Glyoxylate and dicarboxylate metabolism”, “Insect hormone biosynthesis”, “Phenylalanine metabolism”, and “Pyrimidine metabolism” (Figure 7). A total of 8 differentially expressed metabolites and 22 DEGs involved in the different pathways might play an important role in the differences in effect between the habanero-pepper and control diet.

## 3. Discussion

In the present study, we examined the transcriptional and metabolic responses to a habanero-pepper diet in our Drosophila fly model, which harbors organs/tissues that perform equivalent functions to most mammalian organs [21,23]. The integration of transcriptomics and metabolomics reflected changes in genotype and phenotype, thus providing complementary information about genetic alteration, protein synthesis, metabolism, and cellular function [24].

Habanero pepper (*C. chinense*) has the most pungent fruit in the world because of its high capsaicin content and is also considered one of the most important peppers with high market demand because of its aroma and flavor [8]. Bioactive compounds from pepper are known for their analgesic, cardioprotective, pharmacological, neurological, and anti-obesity properties [25]. Capsaicin, as a major active compound from pepper, has numerous beneficial roles in humans [15]. Dietary capsaicin consumption could have a beneficial effect on weight management by activating brown adipose tissue activity and reducing energy intake via appetite and satiety regulation [26]. In Drosophila, capsaicin effects have been described displaying contrasting responses, for instance, Li et al., [27] showed that capsaicin repels Drosophila females from oviposition, meanwhile, Semaniuk et al. [28] reported that chili pepper extends lifespan in a concentration-dependent manner and also confers cold resistance. Our food intake analysis revealed that there was a slight reduction in the food consumption of flies reared on the habanero-pepper diet when compared to control; however, this reduction was not significant indicating that the habanero-pepper diet did not induce a repulsive response in Drosophila flies. Similar results have been reported where wild-type flies, given a choice between capsaicin-laced sucrose and sucrose alone, showed a preference for capsaicin [29]. Thus, the adaptation to facilitate the Drosophila survival and fitness of both parents and offspring to bitter compounds such as capsaicin is still elusive.

In humans, capsaicin activates *TRPV1* response, which plays a critical role in regulating metabolic health for the whole body, including the cardiovascular system, body weight, glucose, and lipid metabolism [30]. We identified an upregulated gene, *iav*, which is an orthologue of *TRPV1* in humans. The gene *iav* is Ca^2+^-permeable and functions as a non-selective cation channel [31]. In Drosophila, *iav* is involved in several processes, including adult walking behavior, negative gravitaxis, and sensory perception of mechanical stimuli [32]. It is predicted to be part of the cation channel complex and is expressed in several structures including the Johnston organ and chordotonal neurons involved in the mechanosensory process to detect different mechanical forces that can affect animal behavior [33]. Thus, *iav* may have a similar function in Drosophila as *TRPV1* in humans, and it was upregulated due to the heat stimuli caused by capsaicin. However, further research is needed to confirm.

Moreover, our DEG analysis also revealed several genes with functional change associated with olfactory and nutrient-related pathways that modulate olfactory responses in flies. Previous studies using microarray analysis have demonstrated that heat treatment affected olfactory responses and feeding behaviors [34,35]. The upregulation of most of the olfactory-related genes such as gustatory receptors, odorant receptors, and odorant-binding proteins suggests that habanero-pepper feeding with high capsaicin content and pungent flavor may alter olfactory perception, thus modifying the appetitive behavioral response in Drosophila.

Drosophila flies also have nutrient-sensing via the gastrointestinal tract, where nutrients interact with receptors on the enteroendocrine cells in the gut, for a physiological regulatory response to modulate feeding behaviors, food intake, metabolism, insulin secretion, and energy balance [36]. Gut endocrine neurons secrete a neuropeptide called limostatin (*Lst*) to control insulin signaling [37]. Flies reared on a habanero-pepper diet showed upregulation of *Lst*, which may indicate a response to nutrient deprivation. Likewise, we also identified an upregulated adipokinetic hormone, *Akh*, principally known for its mobilization of energy substrates, triggering the conversion of stored glycogen and lipids to free energy [38]. Under normal culture conditions, loss of *Akh* function has no effect on development [39]; however, *Akh* neuron signaling is required for starvation-mediated TG breakdown [40]. Upregulation of olfactory-related genes and the *Akh* hormone may have a direct effect on body weight and TG levels, as we observed flies reared on the habanero-pepper diet showed a significant reduction in body weight and TG level as compared with control-diet flies.

Metabolome analysis of adult flies led to the identification of several deregulated metabolites in response to the habanero-pepper diet. For instance, campesterol was the highest upregulated metabolite, followed by sitosterol and stigmasterol, with 6.2-, 5.8-, and 3.4-fold changes in expression, respectively. Sitosterol, campesterol, and stigmasterol are the most important plant sterols from a nutritional standpoint and comprise up to 98% of total sterols in certain seed and vegetable oils [41]. Sterols function as structural components of all membranes and as precursors for the synthesis of steroid hormones in eukaryotic organisms [42]. Drosophila is a known sterol auxotroph and relies on dietary sterols to produce lipid membranes, lipoproteins, and molting hormones [43]. Dietary sterol supplementation increased the lifespan response to dietary restriction in *D. melanogaster* females [44]. Thus, upregulation of sterols in Drosophila may affect hormone precursors and/or developmental effector production and therefore fly development and structure [45]. In contrast, azelaic acid was highly downregulated in response to the habanero-pepper diet, with −5.6-fold change in expression. Azelaic acid participates in taste and olfactory receptors in organs and tissues relevant to metabolism in rats, specifically in the induction of lipolysis in adipocytes, thermogenesis, and promoted fatty acid oxidation in liver [46].

We created a metabolite–disease interaction network showing the metabolite profiles related to human diseases. Although the relation between diseases and metabolites is still limited, metabolic diseases have become highly frequent [47]. Disperse distribution of metabolite nodes suggests that some metabolites may have an important role in causing multiple diseases, whereas some metabolites may serve as specific markers for a few diseases (Figure 3). For instance, the metabolites cholesterol and linoleic acid (both upregulated), and L-tyrosine (downregulated), were associated with schizophrenia. Disturbed cholesterol homeostasis in the brain might increase the risk of many neurodevelopmental disorders [48,49], whereas the level of tyrosine, an essential amino acid, is altered in people with schizophrenia [50]. Moreover, mannitol was found upregulated on flies fed the pepper diet. Mannitol is related to Alzheimer’s disease, lung cancer and ribose-5-phosphate isomerase deficiency. Inhaled mannitol could improve lung function in patients with cystic fibrosis and lung cancer [51,52].

Furthermore, in our transcriptome analysis, we identified genes related to different human diseases. For instance, *Gba1a*, with glucosylceramidase activity involved in adult locomotory behavior in Drosophila, was downregulated in flies that were fed the habanero-pepper diet. Mutations in *GBA1* are among the most common known genetic risk factors for the development of Parkinson’s disease in humans [53,54]. Homozygous *dGBA1b* mutants in Drosophila exhibit shortened lifespan, locomotor and memory deficits, and neurodegeneration [55]. Although reduced *Gba1a* activity leads to the accumulation of glucosylceramide and glucosylsphingosine, these compounds were not found in our metabolome analysis.

Finally, the integration analysis between transcriptomic and metabolomic data at the pathway level provided a visualization of the interactions between DEGs and differentially accumulated metabolites. Data integration revealed six uniquely enriched pathways in response to the habanero-pepper diet. Glycerolipid metabolism, as an integrated metabolism, is responsible for maintaining body temperature by modulating lipolysis via TG breakdown [56]. Moreover, phenylalanine is an essential amino acid that is converted to tyrosine by phenylalanine hydroxylase (*Henna*). The *Henna* gene, phenylalanine, and 3-phenyllactic acid were upregulated in flies on a habanero-pepper diet. Hot pepper fruits are a good source of phenylalanine because its concentration is directly related to capsaicinoids content [57]. In Drosophila flies, phenylalanine, glycine, serine, and threonine metabolism have been related to the aging process. In humans, phenylalanine concentrations were found higher in young than older individuals [58,59]. Glycine and serine metabolism provides the essential precursors for proteins and nucleic acid biosynthesis [60]. Integration of metabolomic and transcriptomic data revealed the dysregulation of the insect hormone biosynthesis pathway with the habanero-pepper versus control diet, which might affect insect metamorphosis in Drosophila [61]. These findings enhance the integration between transcripts and metabolomics and provide deeper insights into the molecular mechanisms involved in the response to dietary habanero-pepper consumption.

## 4. Materials and Methods

### 4.1. Drosophila Stocks and Cultures

All flies used in the experiment were wild-type Berlin-K (8522) genotype *D. melanogaster* (Indiana University, Bloomington, IN, USA. All stocks were maintained on standard cornmeal Drosophila medium in an incubator at 25 °C and 30–50% humidity.

### 4.2. Quantification of Phytochemicals from Habanero Pepper

Capsaicin and dihydrocapsaicin content of habanero pepper used in this study were determined as described by [62], and carotenoid and flavonoid content were estimated as described by [63] with an HPLC system equipped with a 1525 binary HPLC pump, 2707 autosampler, and 2998 photodiode array detector (Waters Corp., Milford, MA, USA).

### 4.3. Experimental Diets: Control and Habanero-Pepper

Populations of the Berlin-K (8522) Drosophila genotype were placed on control and habanero-containing diets. The control diet consisted of autoclave-sterilized standard cornmeal medium (Nutri-fly Bloomington formulation, Genesee Scientific, San Diego, CA, USA) solidified with agar and supplemented with 0.5% propionic acid (*v*/*v*) and 1.5% Tegosept (*w*/*v*) as preservatives. The habanero-pepper diet included this formulation and also 7.5% (*w*/*v*) ground and dried habanero pepper. All experiments and culturing were performed in controlled conditions at 24 °C on a 12-h light/dark photoperiod. Experiments were initiated by placing 10 male and 10 female flies into vials containing the different diets. Adults were allowed to lay eggs for 96 h before being removed. The larvae were fed, and once the adult stage was achieved, these flies were selected for body weight, triglycerides (TG), and glucose measurements; total RNA extraction; and metabolome analysis. Each of the control and pepper diet-reared lines were maintained in three independent replications. Adult flies were placed into Eppendorf tubes in batches per each sex and stored at −80 °C.

### 4.4. CAFE Assay

The amount of food eaten by single flies was measured using the Capillary Feeder (CAFE) assay [64,65]. Briefly, 10 mated flies (5 females and 5 males) were distributed into standard fly vials (34 mm × 100 mm) containing 1% agar at the bottom to maintain internal chamber humidity. Four 5 μL glass capillaries (53432-706, VWR) were inserted into the vial through the lid using 20-μL trimmed pipette tips to hold the capillaries in each CAFE chamber. The capillaries were filled with control and habanero-pepper diet as described above. Capillaries ends were placed at the same level (~4 cm from the lid) in each chamber. The amount of food in the capillaries was measured for 24 h using a caliper. One parallel vial void of flies was used as a control to determine the extent of food evaporation from the capillaries. The total food intake (μL/fly) was calculated as (food consumption evaporation loss)/number of flies. Collected data were analyzed by unpaired, two-tailed *t*-test to detect significant differences among diets.

### 4.5. Body Weight Measurement

After 5 days of the adult fly stage, adult flies were measured for body weight with three biological replicates. The vials containing the flies were first anesthetized by using FlyNap (Item #173010, Carolina Biological Supply Company, NC, USA). Then 10 flies for each sex were separated per replication and weighed individually by using an electronic balance (Mettler Toledo #XS64, Columbus, OH, USA). The weight was measured in milligrams and averaged.

### 4.6. Triglycerides (TG) Measurement

Adult flies were collected in groups of five females and five males and placed in 1.5-mL microcentrifuge tubes. Their live weight was determined, and flies were frozen on dry ice. Flies were then homogenized in 300 µL phosphate buffered saline (PBS) for 1 min at 6000 rpm by using a high throughput ball-bearing homogenizer (Talboys). The homogenates were then centrifuged at 10,000 rpm for 5 min, and 20 µL supernatant was transferred to 200 µL triglyceride reagent (TR22421 Thermofisher, Waltham, MA, USA). The mixture was measured for TG at OD 550 nm by using the SpectraMax M2e instrument (Molecular Devices Corp., San Jose, CA, USA) and the measurement was compared to a standardized curve.

### 4.7. Glucose Measurement

Whole-body total glucose was determined as described by [66]. Groups of five flies per sex were homogenized in 100 µL of 100 mM PIPES buffer (Sigma P6757, St. Louis, MO, USA) with porcine kidney trehalose at 5 µL per 2 mL (Sigma T8778) for 1 min at 6000 rpm by using a high throughput homogenizer (Qiagen, Hilden, Germany). Trehalose converts trehalose (present in the hemolymph) into glucose, so total available glucose levels were measured. The homogenates were then incubated at 37 °C for 1 h, and 10 µL was transferred to 100 µL of the Glucose GO assay kit (Sigma-Aldrich, St. Louis, MO, USA). The reaction mixture was incubated in an Environ Shaker at 37 °C for 10 min, and glucose was measured at OD 340 nm by using the SpectraMax M2e instrument (Molecular Devices Corp.) in relation to deionized water.

### 4.8. Metabolome Analysis

An approximately equal number of adult males and females from each treatment were used for metabolome analysis to limit potential variability based on sex. Metabolite profiles were acquired by using a gas-chromatography mass-spectrometry (GC-MS) system (Agilent Inc., Santa Clara, CA, USA) consisting of an Agilent 7890 gas chromatograph and an Agilent 5975 MSD and 7683B autosampler at the Metabolomics Laboratory of the Roy J. Carver Biotechnology Center, University of Illinois at Urbana-Champaign, USA. The spectra of all chromatogram peaks were evaluated by using the AMDIS 2.71 program (NIST, Gaithersburg, MD, USA) with a custom-built database (460 unique metabolites). The relative quantification of total fly metabolites was determined by normalizing the intensity of the added internal standards during extraction and measured body weights. Metabolites were excluded from analysis if they were undetected in ≥90% of samples in all biological groups. Data visualization and statistical analyses for metabolome data involved using MetaboAnalyst 5.0 [67] and Paintomics 4.0 [68].

### 4.9. Total RNA Extraction and RNA-Seq Library Construction

Total RNA was isolated from the whole-body tissue of flies reared on control and habanero-pepper diets in triplicate. Twenty flies (10 female and 10 male) were selected for each sample and were surface-sterilized with 5% sodium hypochlorite, then total RNA was extracted by using the Trizol reagent (Thermofisher 15596026). Total RNA purification involved using the QIAquick PCR Purification Kit (Qiagen 28104, Hilden, Germany) following the manufacturer’s instructions. Degradation and contamination of total RNA was monitored before RNA library preparation on 1% agarose gels. The RNA quantity and quality were measured by using Qubit 2.0 Fluorometer and 2100 Bioanalyzer (Agilent Technologies, Santa Clara, CA, USA). Samples with RNA integrity number >7 were used for library preparation. An equal amount of RNA (1 μg) was used to construct the RNA-Seq libraries with the NEBNext Ultra RNA Library Prep Kit for Illumina (New England Biolabs, Ipswich, MA, USA) according to the manufacturer’s instructions. In this protocol, mRNA was enriched with Oligo [50] beads from total RNA, and mRNA was fragmented and primed for reverse transcription into cDNA by using random primers. The double-stranded cDNA was purified by using SPRIselect beads and further end-repaired and adaptor-ligated. Libraries were size-selected by using SPRIselect beads, enriched by PCR and quality assessed on a Bioanalyzer. RNA-Seq libraries were pair-end sequenced on an Illumina NextSeq 500 system with 75-bp read length. Sequences were deposited in the NCBI SRA repository under BioProject ID: PRJNA860149.

### 4.10. Sequencing Analysis and Identification of Differentially Expressed Genes (DEGs)

Raw reads were quality checked by using FASTQC and were adapter– and low-quality–filtered (Q value ≤ 30) by using Sickle. Clean reads were aligned to the Drosophila genome (http://ftp.flybase.net/releases/FB2020_05/dmel_r6.36/fasta/ accessed on 1 July 2021) by using BWA-MEM [69]. Reads mapping to each gene feature were counted by using HT-Seq to create a raw gene count table for annotated genes identified with at least one mapped read for the Drosophila genome. Differential expression analysis involved using iDEG [70] by comparing samples for the control and habanero-containing diets. The expression patterns of transcripts were investigated by absolute Log2 ratio ≥ 2 and false discovery rate ≤ 0.05. Blast2go [71] was used to determine Gene Ontology (GO) terms enriched in DEGs.

### 4.11. Statistical Analysis

All data in this study are expressed as mean ± SD from three biological replicates. ANOVA was used to detect significant differences in body weight and TG and glucose levels.

## 5. Conclusions

In conclusion, our results demonstrate altered pathways at the transcriptome and metabolome level in flies reared on a habanero-pepper diet. Habanero pepper affects sensory abilities in *D. melanogaster*, such as olfactory-driven behaviors and olfactory sensitivity to several odorants, which in turn changes the molecular levels of some genes. A better understanding of the complex mechanisms that underlie olfactory modulation might help in characterizing the olfactory systems that are affected by habanero-pepper consumption. This study also revealed the relation between different metabolites and genes related to human diseases. However, further functional characterization with mutant analysis is needed to confirm their role in response to a habanero-pepper diet.

## Figures and Tables

**Table 1:** Selected genes with differential expression in response to habanero-pepper diet and their orthologues in humans.

Gene Name	Gene Symbol	Differential Expression	Annotation	Human Orthologue	Function in Humans
FBgn0086693	*iav*	[UP]	Encodes a protein involved in the response to different stimuli such as startle, heat, or sound	*TRPV1*	*TRPV1* Activation in response to heat
FBgn0036181	*Muc68Ca*	[UP]	Expressed in embryonic/larval midgut; embryonic/larval salivary gland	*Muc2*	Protective role for gastrointestinal tissues
FBgn0053300	*Muc30E*	[UP]	Expressed in amnioserosa; embryonic/larval salivary gland		
FBgn0031273	*CG2839*	[DOWN]	Predicted to enable signaling receptor activity	*Reg3g*	Modulate intestinal hormones and microbiome
FBgn0051661	*CG31661*	[DOWN]	Predicted to enable aspartic-type endopeptidase activity	*PGC1-*α	Fatty acid metabolism
FBgn0004552	*Akh*	[UP]	Adipokinetic hormone (Akh) encodes a peptide hormone secreted by the corpora cardiaca		Lipid metabolism
FBgn0263593	*Lpin*	[UP]	Encodes a protein that plays a central role in fat body function and energy metabolism	*Lpin2*	Lipid and energy metabolism
FBgn0033782	*sug*	[DOWN]	Encodes a transcription factor that regulates the expression of insulin-like peptides and genes involved in lipid and carbohydrate metabolism		
FBgn0034140	*Lst*	[UP]	Encodes a peptide hormone produced by endocrine corpora cardiaca cells during starvation		
FBgn0287184	*FASN3*	[DOWN]	Fatty acid synthase	*FASN*	Fatty acid metabolism
FBgn0051148	*Gba1a*	[DOWN]	Enables glucosylceramidase activity, involved in adult locomotory behavior	*GBA*	

UP, upregulated; DOWN, downregulated.

## Data Availability

Sequences were deposited in the NCBI SRA repository under BioProject ID: PRJNA860149.

## Supplementary Materials

The following supporting information can be downloaded at: https://www.mdpi.com/article/10.3390/ijms23179924/s1.
