# Supplementary material for: Peppers in Diet: Genome-Wide Transcriptome and Metabolome Changes in Drosophila melanogaster
Source: Int J Mol Sci. 2022 Sep 1;23(17):9924. doi: 10.3390/ijms23179924 (PMC9455967; doi:10.3390/ijms23179924)
Supplement: Supplementary file 1 [file ijms-23-09924-s001.zip › Supplementary Figures.pdf]

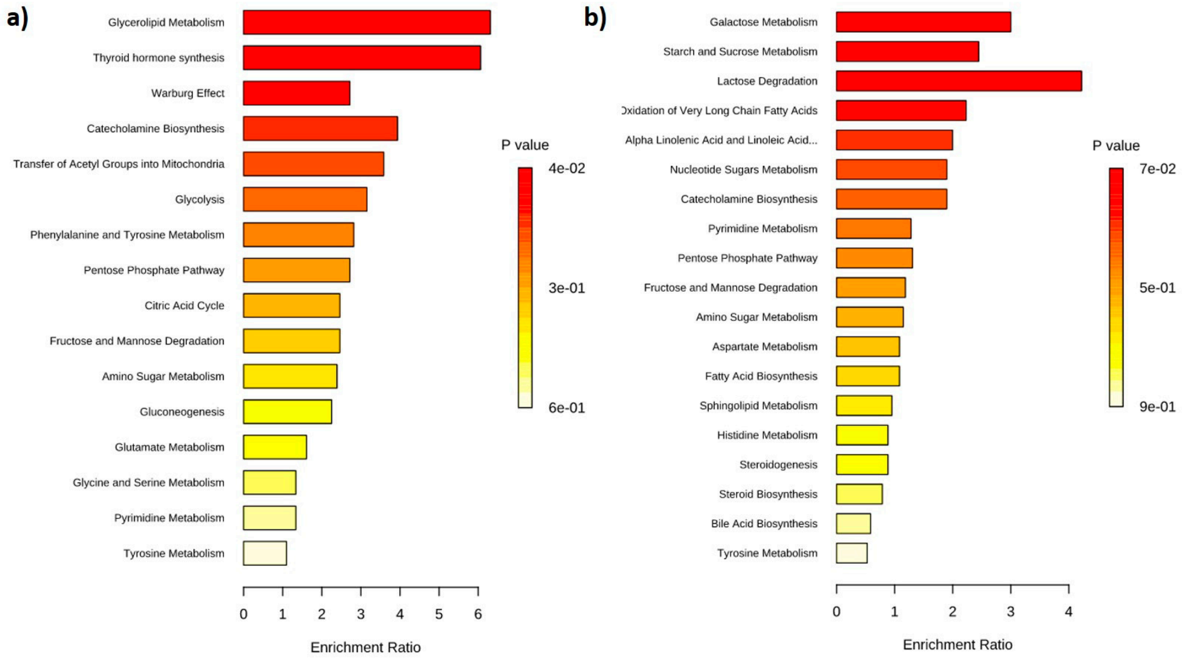

Figure S1. KEGG pathway enrichment comparison of significant upregulated (a) and downregulated (b) metabolites in *Drosophila* in response to a habanero-pepper diet. The y-axis indicates the different term categories, the x-axis the rich factor and the color of the bars the p-value of enrichment analysis.

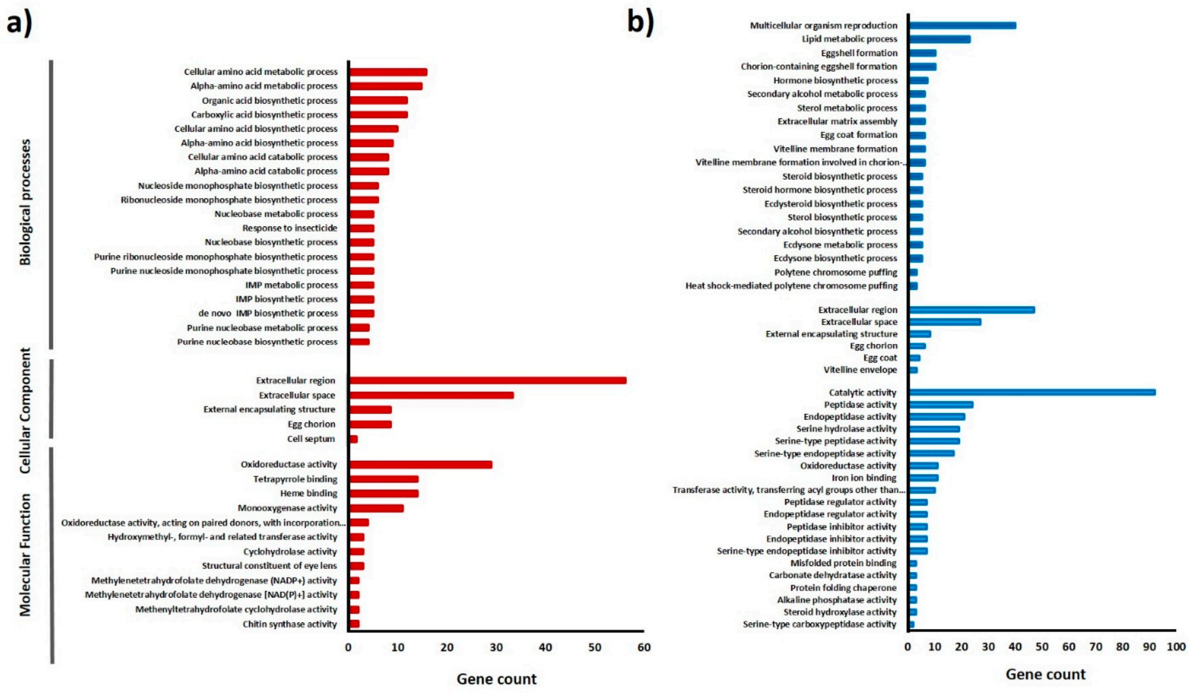

Figure S2. Gene Ontology (GO) classification of the differentially expressed genes. The figure shows partial GO enrichment for the upregulated (a) and downregulated (b) genes in three categories: biological process, molecular function and cellular component.
